# An important step towards a prevascularized islet microencapsulation device: in vivo prevascularization by combination of mesenchymal stem cells on micropatterned membranes

**DOI:** 10.1007/s10856-018-6178-6

**Published:** 2018-11-09

**Authors:** Milou Groot Nibbelink, Katarzyna Skrzypek, Lisanne Karbaat, Sanne Both, Jacqueline Plass, Bettie Klomphaar, Jéré van Lente, Sieger Henke, Marcel Karperien, Dimitrios Stamatialis, Aart van Apeldoorn

**Affiliations:** 1 Developmental BioEngineering, MIRA Institute of Biomedical Technology and Technical Medicine, University of Twente, Maastricht, The Netherlands; 2 (Bio)artificial organs. Department of Biomaterials Science and Technology, MIRA Institute of Biomedical Technology and Technical Medicine University of Twente, Maastricht, The Netherlands; 3 Biomedical Signals and Systems, MIRA Institute of Biomedical Technology and Technical Medicine, University of Twente, Maastricht, The Netherlands; 4 Present Address: Complex Tissue Regeneration, MERLN Institute for Technology Inspired Regenerative Medicine, Maastricht University, Maastricht, The Netherlands

## Abstract

Extrahepatic transplantation of islets of Langerhans could aid in better survival of islets after transplantation. When islets are transfused into the liver 60-70% of them are lost immediately after transplantation. An important factor for a successful extrahepatic transplantation is a well-vascularized tissue surrounding the implant. There are many strategies known for enhancing vessel formation such as adding cells with endothelial potential, the combination with angiogenic factors and / or applying surface topography at the exposed surface of the device. Previously we developed porous, micropatterned membranes which can be applied as a lid for an islet encapsulation device and we showed that the surface topography induces human umbilical vein endothelial cell (HUVEC) alignment and interconnection. This was achieved without the addition of hydrogels, often used in angiogenesis assays. In this work, we went one step further towards clinical implementation of the device by combining this micropatterned lid with Mesenchymal Stem Cells (MSCs) to facilitate prevascularization in vivo. As for HUVECs, the micropatterned membranes induced MSC alignment and organization in vitro, an important contributor to vessel formation, whereas in vivo (subcutaneous rat model) they contributed to improved implant prevascularization. In fact, the combination of MSCs seeded on the micropatterned membrane induced the highest vessel formation score in 80% of the sections.

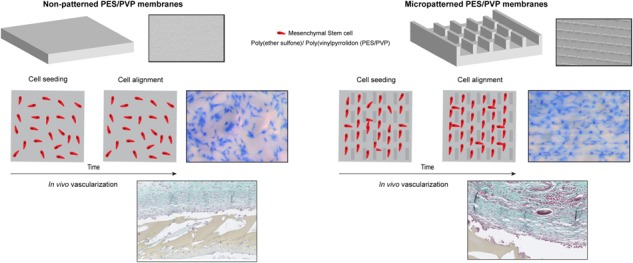

## Introduction

Type 1 Diabetes mellitus is a chronic disease that manifests in children and young people (usually <30 years). An autoimmune reaction destructs the insulin producing β cells resulting in hyperglyceamia as well as relative insulin deficiency [1–5]. Diabetes Type I is known for its severe acute and long-term complications due to micro- and macroangiopathic lesions and has a significant social and economic impact. Long term symptoms are retinopathy, neuropathy, and nephropathy [4, 6–12].

Due to the inadequate produced insulin, type 1 Diabetes mellitus patients need life-long insulin therapy and tight glucose monitoring. Patients with severe glyceamic lability, recurrent hypoglycaemia, hypoglycaemia unawareness, or an insufficient response to the insulin therapy are in need for alternative therapies. Current alternative treatments are total pancreas transplantation or clinical islet transplantation [4, 7, 8, 11, 12]. Both alternatives have the disadvantage of limited donor availability and a need for life-long immunosuppressive drugs as both the pancreas and islets are of allogeneic origin. The advantages of islet transplantation over whole pancreas transplantation are the lower surgical risk and fewer complications [8].

In CIT 60-70% of the donor islets of Langerhans are lost immediately after transplantation. This is due to many different factors including mechanical stress, different immune-responses, and lack of vascularization. In fact, after intraportal infusion, islets are immediately exposed to high concentrations of drugs and nutrients, such as glucose, which negatively affects their function [13–20]. Additionally, the islets are in a pro-inflammatory state at the moment of transplantation due to the isolation procedure, therefore, they express inflammatory mediators, leading to the onset of different immune-responses, like Instant Blood Mediated Immune Response (IBMIR) and alloresponse which in the end leads to graft failure [21].

Previous research has focused on improving the transplantation outcome by immune-protective strategies that prevent immune cells from reaching encapsulated islets while maintaining islet viability. Good examples of this are membrane based scaffolds as they could maintain islet viability and act as a physical barrier for the immune system. These scaffolds should meet stringent requirements: islets need to be separated from the blood stream, the device needs to be permeable for glucose, insulin, nutrients, and oxygen, and the device needs to be impermeable to the immune cells [7, 14, 22–26].

One of the key issues related to the development of an immune protective scaffold for extrahepatic islet transplantation is the scaffold prevascularization or the enhanced vascularization directly after implantation. It is actually important to provide blood supply close to the islets since the isolation process disrupts their own vasculature whereas the islets are normally highly vascularized in the pancreas. In fact, islets receive 5-15% of the total blood supply of the pancreas while they only consist of 1% of the entire pancreas mass [6, 27, 28]. It is known that hypoxia leads to a loss of viability and glucose responsiveness. Neo-angiogenesis will allow perfusion of islets, however, this generally only starts approximately 7 days post transplantation. It is obvious that enhancing vascularization around the implant would be critical to optimal islet survival and function [7, 13–15, 22, 29].

Improved implant vascularization would also reduce the inflammatory response during first post-transplantation period. Due to better vascularization, higher oxygen supply will be available thereby reducing hypoxia in islets. Normally, hypoxia results in islet ischemia followed by the production of reactive oxygen species (ROS). Resulting in an activated inflammatory pathway NF-kB [13, 30].

There are many different ways to enhance vascularization of the encapsulated islets, either by prevascularization of the device or by induction of vascularization in vivo [28, 31]. This can be achieved by the release of angiogenic factors by drugs or cells. Materials with specific surface topographies can also enhance blood vessel formation and reduce the immune response that is responsible for the formation of the fibrous capsule [26, 32]. Already in 1990’s, Baxter Healthcare discovered that biomaterial topography could positively influence vessel formation [32]. Moreover, when designing surface topographies for vessel formation, an important factor would be the induction of cell alignment, as it has been shown that cell alignment induces vascularization [33].

Moreover, the islet implant vascularization could also be improved by culturing cells with endothelial potential, like mesenchymal stromal cells (MSCs), fibroblasts, endothelial (progenitor) cells, and bone marrow progenitor cells. This can be achieved by either seeding cells directly on the scaffold or by coating the islets with cells [26, 34–41]. Besides, the MSCs do not only contribute to the induction of vessel formation, but they could also reduce the immune response against the encapsulated islets. Therefore, autologous MSCs, derived from a bone marrow aspirate taken from the patient may offer a big advantage for clinical islet transplantation [42–46].

**Fig. 1:**
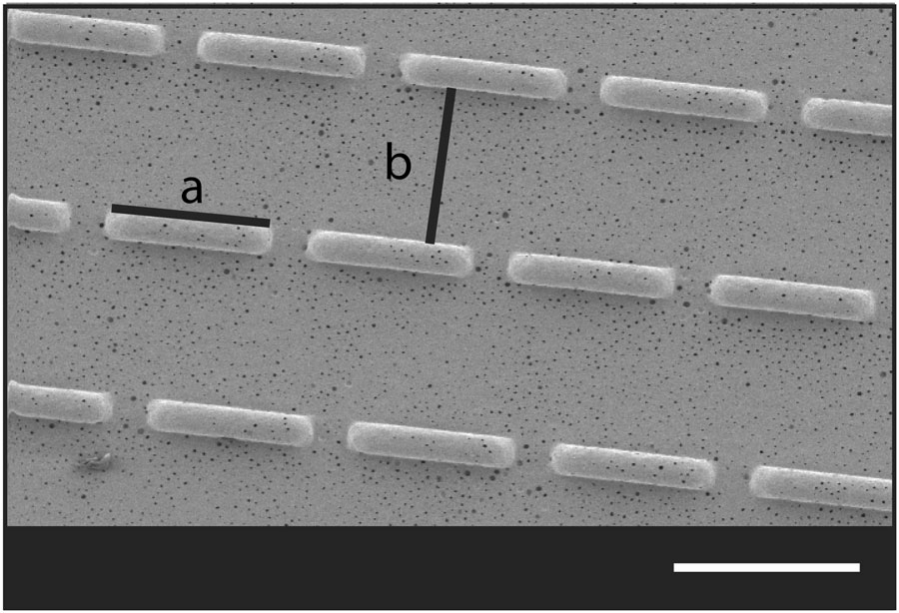
Micropatterns porous membrane with brick like surface topographies. The bricks have height of 40 μm and length of 100 μm **a**. The spacing between the bricks pattern is 100 μm **b**

In our recent work, we showed the development of an encapsulation device for islets of Langerhans. The device consisted of a microwell poly (ether sulfone)/polyvinyl pyrrolidone (PES/PVP) membrane for islet separation covered by a membrane lid. Both membranes were porous to allow nutrients, glucose, and insulin to diffuse through [47]. In a recent study, we also reported that a micropatterned lid can induce alignment of human umbilical vein endothelial cells (HUVEC) [48]. In fact, a surface topography of an intermittent brick pattern there allowed for communication between cells and the connection of HUVEC branch-like structures creating a network over the membrane surface (Fig. 1). This was achieved by co-culture of HUVECs on the monolayer of fibroblasts without the addition of hydrogels. There, we coated the membrane lid with fibronectin but we did not use angiogenic gels since those could block the membrane pores limiting the transport properties of the implant. The distance between the patterns was 100 µm, since it has been shown that cell alignment occurs in patterns between 20–130 µm [49]. These results were in accordance with literature where it has been shown that HUVEC alignment can be tailored using micropatterns [50–52].

In this work, we investigate whether these micropatterned membranes with brick like surface topography could also induce MSC organization in vivo. Cell attachment to the membrane was achieved using a fibronectin coating and the membranes were implanted subcutaneously in a rat model. To the best of our knowledge, this study is the first that investigates the combined effect of micropatterned membranes and MSCs for inducing vascularization of an implant device without the addition of hydrogels or angiogenic factors.

## Materials and Methods

### Membrane fabrication

A 15 wt% poly(ether sulfone) (PES) (Ultrason, the Netherlands) 5 wt% poly-(vinylpyrrolidon) (PVP) (Sigma-Aldrich) polymer solution in N-methylpyrrolidone (NMP) (Sigma-Aldrich) was used to cast membranes as described by Skrzypek et al. [48]. In short: The membranes were cast on a glass plate (for non-patterned membranes) or on a micropatterend silicon wafer for the brick like surface topography. The micropatterns were 40 μm in height and 100 μm apart and the bricks had a length of 100 μm (Fig. 1). When, directly after casting, the polymer was submerged in a water coagulation bath, phase separation occurred and porous membranes were formed. Finally, all the membranes were rinsed with demineralized water to remove remaining solvent traces and stored in demineralized water till further use.

### Animal housing and cell culture

The ethical committee of the University Medical Center Utrecht approved the animal experiments. The care and operative procedure of the rats were performed following the regulation of the central laboratory animal institute in Utrecht. All animal studies were performed at the University of Twente. Rat bone marrow derived MSCs were harvested from two 10-11-week-old female Lewis rats (Harlan, the Netherlands). Rats were euthanized with CO_2_, shaved, and the skin was sterilized using 70% ethanol. Both femurs were resected and placed in sterile PBS with 200 U/mL Penicillin and 200 mg/mL Streptomycin (Gibco) for at least 15 min The epiphysis was cut off and the femur was flushed with Minimum Essential Mediumα (α-MEM, Life Technologies) with 2 mM GlutaMAX, 100 mU/mL penicillin and 1mg/mL streptomycin (Gibco), and 0.2 mM L-ascorbic acid-2-phosphate (ASAP) using a 22G needle and syringe. The cell suspension per femur was plated in 1 flask. FBS was added to the cell suspension with a final concentration of 10%. After three days, the medium was refreshed. MSCs were grown to 80% confluence using Minimum Essential Mediumα (α-MEM, Life Technologies) with 10% FBS, 2 mM GlutaMAX, 100 mU/mL penicillin and 1mg/mL streptomycin (Gibco), and 0.2 mM L-ascorbic acid-2-phosphate (ASAP).

### Coating of membranes

The porous membrane was coated with fibronectin (Sanquin, Amsterdam) to enable cell attachment, as described elsewhere [48]. A fibronectin solution of 200 μg/ml was prepared in PBS. The solution was poured on the membranes and incubated for 30 min at 37 °C. After this, the membranes were incubated with culture medium for 1 h, after which cells were seeded on top.

### Cell attachment on membranes

MSC attachment on flat porous PES/PVP membranes uncoated or coated with fibronectin (200 µg/mL) was assessed, as described in Skrzypek et al. [48]. In short: MSCs were seeded with a density of 10.000 cells/cm^2^ and kept in culture for 1 day. Subsequently, samples were fixed in 10% buffered formalin for 10 min Subsequently, samples were fixed in 10% buffered formalin for 10 min After fixation samples were washed in dH2O (2x) and stained with methylene blue (Sigma-Aldrich) for 10 sec. followed by a 3x wash with dH2O. Quantification of the number of cells on the membranes was done by taking 3 pictures of each membrane and counting the number of cells (Nikon SMZ800 microscope).

### Cell alignment on micropatterned membranes

MSCs were seeded on micropatterned membranes to assess cell alignment following the surface topography (n=3). Similar to the cell attachment experiments, the cells were seeded on 200 µg/mL fibronectin coated membranes (10.000 cells/cm^2^). After 7 days, the samples were fixed in 10% formalin for 10 min and stained using DAPI (Invitrogen, 1:100). Images were taken using a BDpathway 435 microscope and analyzed using CellProfiler (v 2.1.1). The orientation of the cell nucleus was estimated as: the angle between the x-axis (aligned with the image) and the major axis of the ellipse of the nuclei.

### Micropatterned membranes and MSCs for in vivo vascularization in female Lewis rats

In vivo vascularization of the micropatterned membranes and the addition of MSCs were tested by subcutaneous implantation in female Lewis rats. In short; non-patterned and patterned PES/PVP membranes (0.79 cm^2^) were sterilized in 70% ethanol, washed in PBS and coated with 200 µg/mL fibronectin. MSCs isolated from female Lewis rats were seeded on the membranes (Pooled from 4 femurs, Passage 2, 3.000 cells/cm^2^). After 4 days of culture membranes were ready for implantation. Control membranes were fixed and stained for methylene blue to confirm the presence of a monolayer upon implantation. Eleven to twelve -week-old female Lewis rats (n=6) (Harlan, the Netherlands), were injected subcutaneously with Carprofen (5 mg/kg) 30 min before surgery. Then, they were anesthetized using isoflurane, their back was shaved and sterilized using 70% ethanol and Betadine. During the entire surgery, their temperature was monitored rectally. Six subcutaneous pockets were created on the back of each rat. In each pocket, a membrane was implanted. Each condition was implanted in each rat in a randomized order to correct for a possible influence of location. The incisions were first closed intracutaneously and finally the skin was sutured. After 14 days, the rats were euthanized with CO_2_, samples were explanted, fixed in 4% buffered paraformaldehyde (ON, 4 °C, Sigma Aldrich), and processed for immune-histochemistry. From each sample three locations with at least 125 μm between the samples were used for analysis. Sections were stained for Toluidine Blue to detect mast cells. In short Toluidine blue (Sigma Aldrich) was dissolved in 70% ethanol. At the day of staining, the stock solution was 10x diluted in 1% sodium chloride, after which the pH was set at 2.3. Deparaffinized and hydrated sections were stained with the working solution for 3 min Finally, the samples were dehydrated, cleared with xylene and mounted in Tissue Tex. A Masson Goldner Trichrome staining was performed to detect vessels using the manufacture’s protocol (Merck Chemicals, Darmstadt, Germany). Stained microscopy slides were scanned using a Nanozoomer slide scanner 2.0 RS (Hamamatsu, Hamamatsu City, Japan). Trichrome stained sections from day 14 samples (with a folded configuration upon explantation) were scored by three blinded individuals, independently to assess the extent of vessel formation. Sections were scored with – (no blood vessels), + (1-2 blood vessels), or ++ (> 3 blood vessels) for vessel formation around the implant.

### Statistical analysis

To determine the effect of fibronectin concentration on cell attachment the following analysis was performed. From each sample three pictures were taken, since all used cell types have the tendency to grow in clusters after initial attachment, pictures were taken of the densest, least dense and average covered areas. Average cell numbers on 1 mm^2^ were determined for each sample. Statistical differences in cell numbers between the conditions and control were determined by a Welch t-test (P<0.05 = *, P<0.01 = ** and P<0.001 = ***). Fleiss’kappa was used to establish agreement between the three observers of the Trichrome staining. The overall obtained kappa was 0.32 (fair agreement) and there was a moderate agreement (κ = 0.63) for the ++ classification.

## Results

### Attachment and alignment of MSCs to PES/PVP porous membranes

**Fig. 2:**
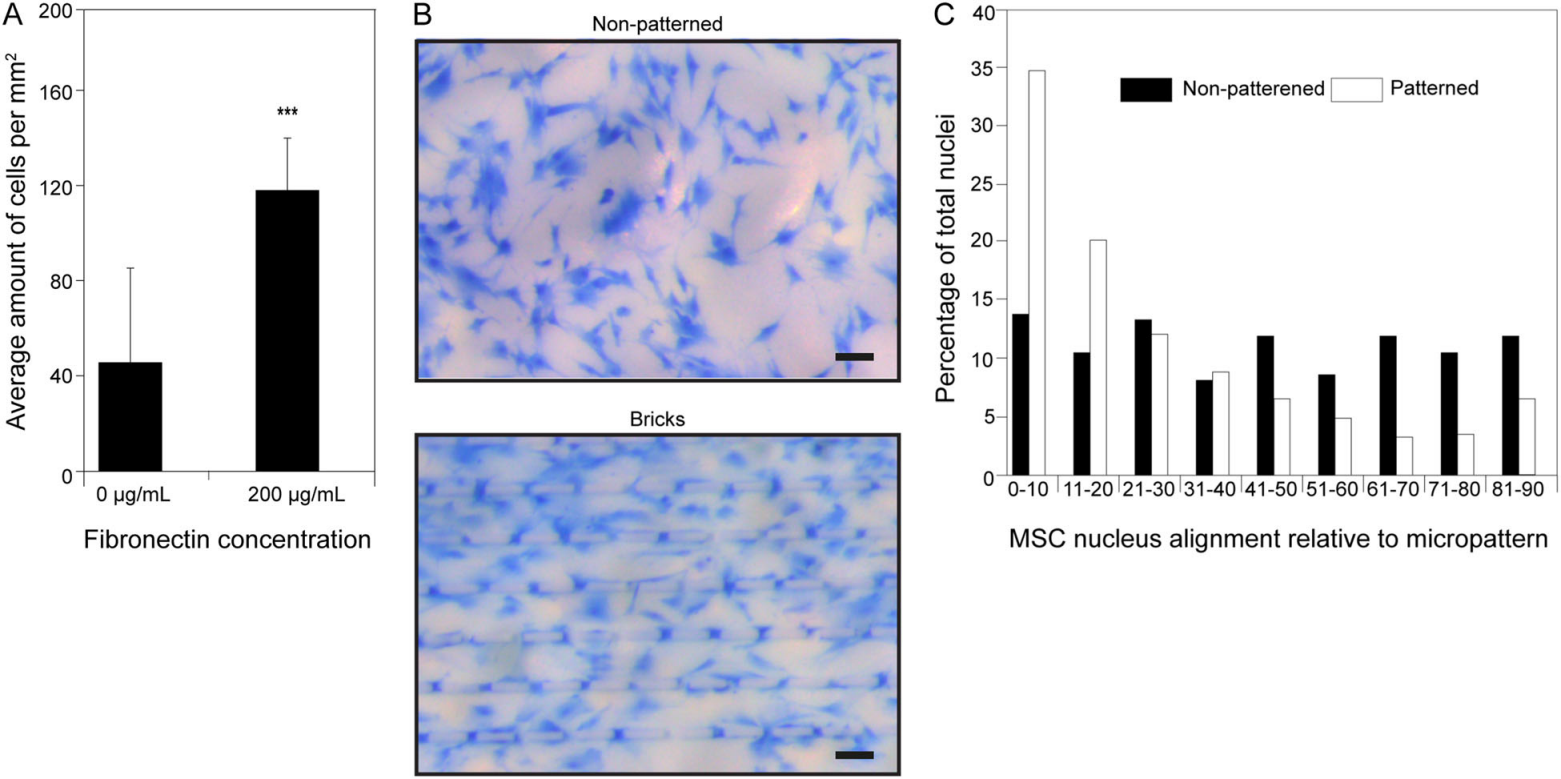
**a** Attachment of MSCs to PES/PVP porous membranes, either uncoated (0 µg/mL) or coated with fibronectin (200 µg/mL). MSCs were seeded (10.000 cells/cm^2^) on to the membranes coated with fibronectin and after 1 day of culture the total number of cells per mm^2^ was determined. **b** Methylene blue staining of MSCs on non-patterned and micropatterned membranes. **c** MSC nucleus alignment relative to micropatterns, where 0 degrees means that the nucleus was oriented parallel to the micropattern. Alignment to flat (black) and micropatterned (white) membranes was determined. Error = SD. Scale bars 100 μm

The PES/PVP membranes have low cell adhesion properties. In fact, the combination of the hydrophobic PES with the hydrophilic PVP is ideal to minimize the protein adhesion and thereby cell adhesion. Therefore, PES/PVP is the main biomaterial for the fabrication on membranes for blood filtration where low cell adherence and fouling is required [53]. Previously, we found that a fibronectin coating could achieve good adhesion of fibroblasts and HUVECs on the micropatterned PES/PVP membranes, which is in accordance to other studies [48, 54, 55]. Here, we show, in agreement with Skrzypek et al. [48]. that 200 µg/mL fibronectin coating also improved the attachment of MSCs to PES/PVP membranes (Fig. 2a). Besides, as alignment, spreading and organization of cells increases vessel formation [33], we assessed whether the MSCs would then align to the micropatterns. Fig. 2b shows cell spreading on the PES/PVP membranes. MSCs spread over the surface of both, pattern and non-pattern membrane. Additionally, in case of cells cultured on patterned membranes it was observed that cells spread following the directions of the surface structures. Further analysis of cell nucleus confirmed that the micropatterned membranes induce MSC alignment in contrast to the non-patterned membranes where the MSCs have random orientation and cells are equally distributed in all directions (Fig. 2c).

### In vivo vascularization in female Lewis rats

We also assessed the effect of MSCs cultured on patterned membranes on improving vessel formation in vivo.

**Fig. 3:**
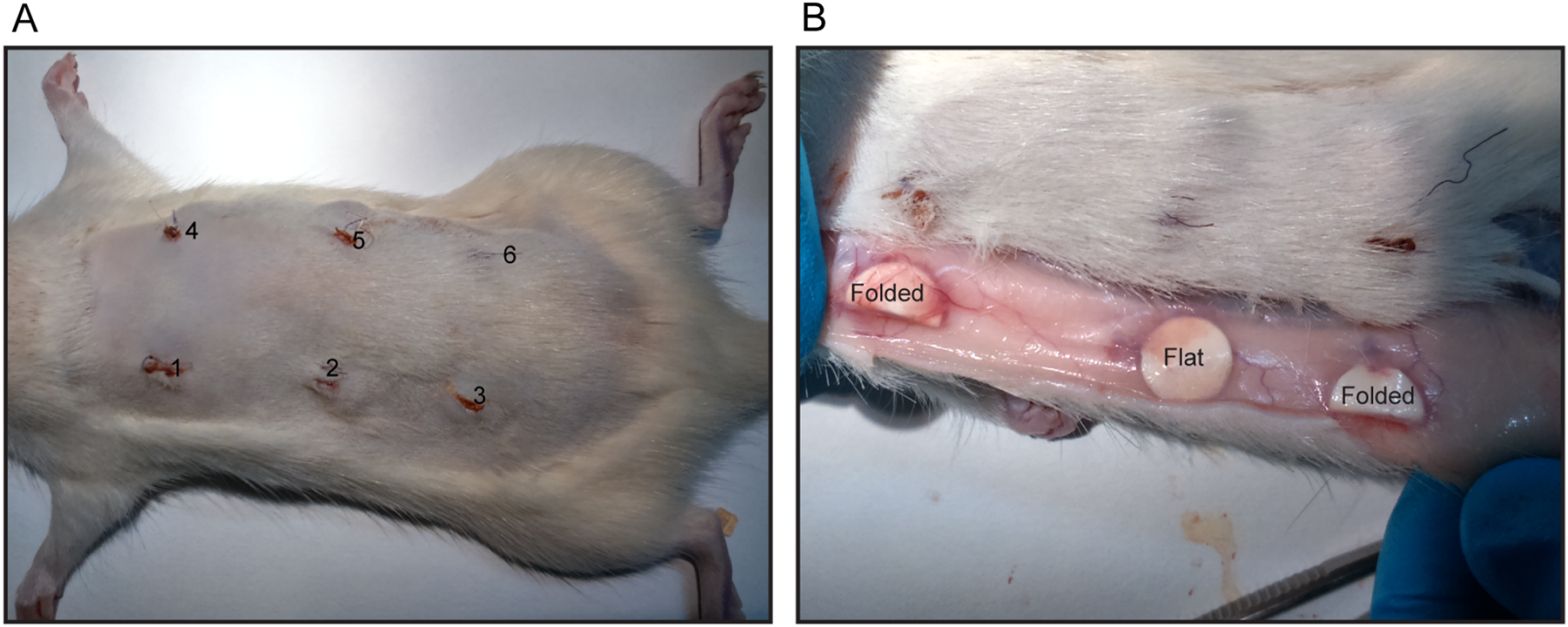
**a** On the left, an example of the six implantation pockets at day 1, on the right **b** different configurations of the membranes upon explantation (Folded and Flat)

Figure 3a shows the six pockets on the back of a rat (left) and different configurations upon explantation (right). After 14 days of implantation, the majority of the membranes (64% of the samples) was explanted easily. Although the samples were implanted flat (Fig. 3b), some were found to be folded upon explantation. Besides, most of the samples that were flat during explantation had less tissue around them compared to the samples that were folded in vivo. Only 45% of the samples were still in a flat configuration upon explantation. Only 25% of these flat membranes were difficult to explant, whereas 45% of the folded samples were difficult to explant due to the presence of more dense tissue surrounding the implant.

**Fig. 4:**
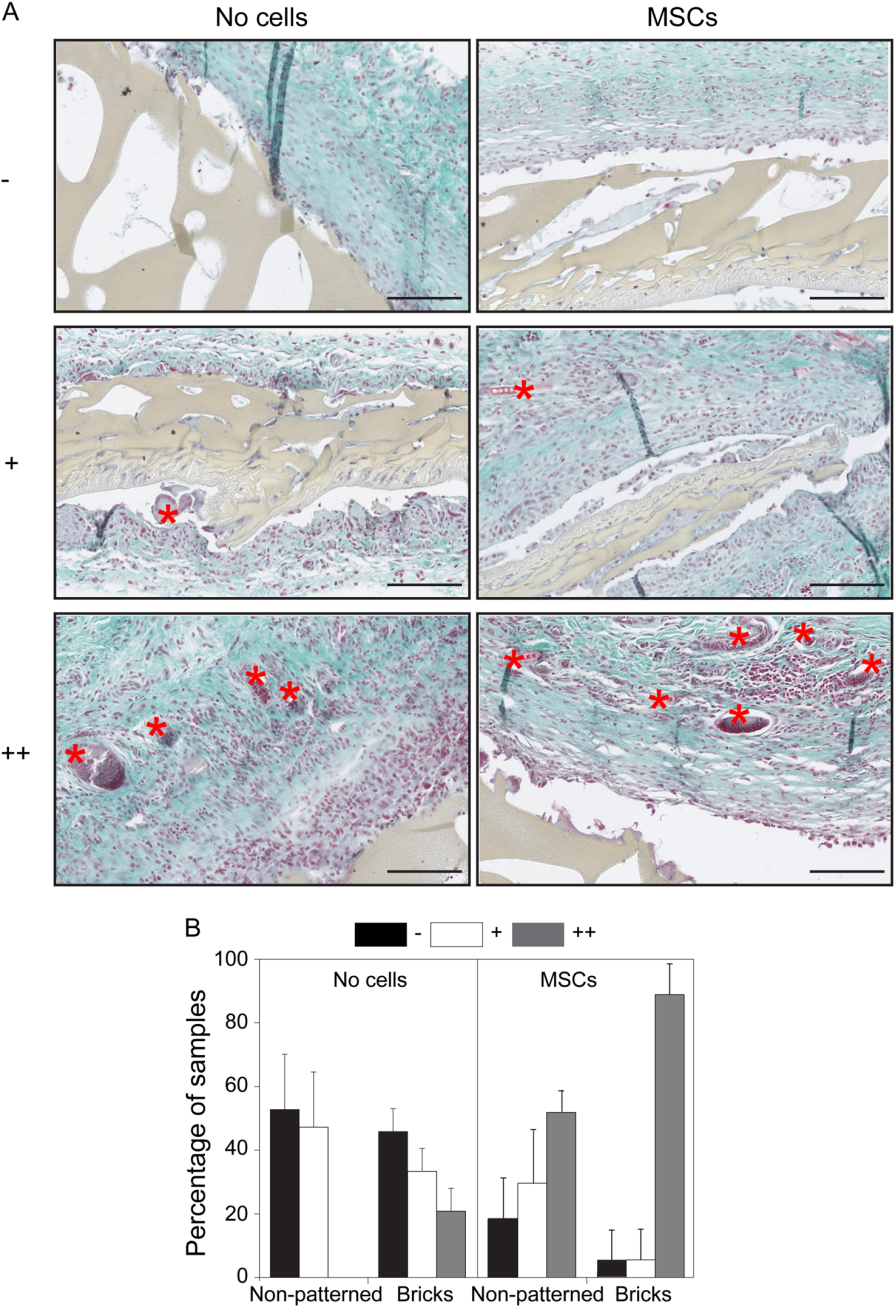
In vivo vascularization in female Lewis rats. **a** examples of Trichrome stained sections of micropatterned membranes. The left (right) panel shows the samples without (with) MCSs cells. From each case, an typical example of each classification is depicted; − top, + middle, and ++ bottom. Examples of vessels are indicated with a red star. Scale bars 125 μm. **b** Analysis of vessel formation in vivo. The samples are classified in three categories; no vessels (− black), some vessels (+ white), and lot of vessel infiltration (++ grey). The effect of non-patterned and micro-patterned membranes on vessel formation in samples without cells and in samples with MSCs. Error = SD

A Masson-Goldner Trichrome staining was used to determine the vessel formation on day 14 for these samples. Vessel formation was independently scored by 3 blinded observers. In fact, the samples were categorized into three groups based on the vessel densities, ranging from samples with no vessels (-), samples with 1 to 2 vessels (+), or samples with more than 3 vessels (++). Figure 4a shows representative pictures of Trichrome stained sections with different vessel densities (from top till bottom) for samples without cells (left panel) and with MSCs (right panel) all with the micropatterned membranes (examples of vessels are indicated with a red star). Representative pictures of the non-patterned membranes are shown in supplementary figure 1. The results of the vessel scoring of the staining are depicted in Fig. 4b. The left part of the graph shows very clearly that without cells, vascularization is increased when micropatterned membranes were used compared to non-patterned membranes. In approximately 20% of all sections with micropatterned membranes, more than 3 vessels (score: ++) were observed, whereas, none of the non-patterned membranes were scored with ++. The addition of MSCs to both membranes resulted in an increased vessel formation compared to these membranes without cells (right and left panel of the graph). In the case, 51% of the non-patterned membranes which were cultured with MSCs had more than 3 vessels (score: ++). Interestingly, the highest vessel formation was observed for the samples where MSCs were cultured on the micropatterned membranes. In fact, 80% of these samples had more than 3 vessels (score: ++), clearly indicating that the culture of MSCs on the micropatterned membranes is necessary for achieving vessel formation in vivo.

## Discussion

Survival of islets of Langerhans encapsulated in an immune-protective device is often hampered due to limited delivery of oxygen and nutrients to the cells as a result of lack of vascularization. After islet isolation and encapsulation, the pancreatic islets are separated from their native vasculature and have no direct access to the blood supply. Since the islets require high blood supply; in fact, although only 1% of the pancreas consists of islets, they receive 5-15% of the total blood supply to the pancreas [6, 27, 28], it is important that one improves vascularization of the implant prior to implantation.

Many different strategies have been proposed to enhance vascularization of the implants; by using angiogenic factors, culture of MSCs or endothelial cells, or application of micropatterned scaffolds [26, 34, 36, 39–41]. In a previous study, we showed that a well-organized interconnected branched structure of HUVECs can be created on a layer of fibroblasts cultured on micropatterned membranes with brick like surface topography [48]. Those membranes can be used as a lid on an islet encapsulation device [47]. Here, we show for the first time that culture of MSCs on these micropatterned membranes can lead to formation of blood vessels around the membrane, in vivo.

The effect of micropatterned membranes and MSCs on vascularization was assessed by implanting PES/PVP membranes subcutaneously in female Lewis rats. Due to low cell adhesion on these membranes, fibronectin coating was applied there to enhance MSC attachment, following earlier coating protocols developed for the attachment of fibroblast cells on these membranes [48]. It is well known that MSCs attach well to fibronectin via RGD binding integrins, and more specifically αv-containing integrins [56–58]. Cell adhesion via integrins to ECM molecules, like fibronectin, activates intracellular signaling pathways that direct cell viability, proliferation and differentiation [59, 60]. Culture of MSCs with ECM molecules such as fibronectin can also improve cell proliferation [61]. Indeed, in our study, the fibronectin coating of the PES/PVP membranes, accelerated the attachment of MSCs significantly in the first 24 h after cell seeding.

In the in vivo study, membranes were coated with fibronectin as this was needed for MSCs attachment. As it is known that fibronectin may enhance vascularization by itself [62, 63], all samples were coated with fibronectin to make sure that observed difference between the non-patterned and patterned membranes was not due to applied coating but due to introduced surface topography.

After 14 days of implantation the membranes cultured with MSCs had better vessel recruitment than the membranes implanted without cells, consistent with other studies [40, 64, 65]. Moreover, induction of vessel formation by MSCs has been shown to directly improve islet graft function [66, 67] and have immunomodulatory effects [68]. In fact, Figliuzzi et al. showed that only transplantation of 2000 islets in combination with MSCs resulted in normogliceamia in rat animal model [66].

In this study, we observed a significant increase in vessel formation around the micropatterned membranes. Actually, around 20% of the patterned samples were scored with 3 vessels or more whereas the non-patterned membrane had very low score. Other studies have shown that surface topographies are able to influence cell behavior, however, the effect is dependent on the dimensions and the specific morphology of the surface topographies applied [49, 69]. For example, Song et al showed that 10 μm micropost-textured PDMS scaffolds, combined with biochemical stimulus and MSCs, had a positive effect on vessel formation in vivo. However, their scaffolds with only surface topographies exhibited no vascularization [69]. In contrast, our membranes with brick like surface topography seem to induce some blood vessel formation without other stimuli, probaby due to the improve cell alignment and the ability of cell communication between the patterns. This was also clearly shown in our recent study of co-culturing HUVECs and fibroblasts on these micropatterned membranes [48]. There, the fibroblasts aligned very well and the HUVECs could span in between the patterns and create branch like cells networks.

A very important finding of this work is the additive effect of MSCs and surface topographies for achieving vessel formation. MSCs seeded on the micropatterned membranes with brick like surface topographies induced the highest vessel formation score (++) in 80% of the samples. Other studies have also combined surface topographies with MSCs however, they were also combined with biological stimuli like VEGF and matrigel [70]. Our study is the first one which reports good vessel formation for the combination of micropatterned membranes with MSCs without the additional of other stimuli. We think that this is probably due to optimal brick like design which induces cell orientation and at the same time achieves good cell communication [48, 71].

## Conclusion and outlook

Survival of islets of Langerhans encapsulated in an immune-protective device is often hampered due to limited delivery of oxygen and nutrients to the cells as a result of lack of vascularization. It is therefore highly important to induce vessel formation after transplantation. In this study, we investigated whether micropatterned membranes and MSC either separately or combined can improve vessel formation in vivo. Our results showed that we can achieve the best results (high vessel formation score (++) in 80% of the samples) when combining MSCs cultured on brick like surface topographies.

In the future, we plan to perform implantation studies of our islet encapsulation device [47] with MSCs on a brick like patterned surface and investigate whether the islet viability and function can be maintained due to vessel formation around the device. Besides, since there are reports that the membrane surface topography and MSCs can also reduce the immune response to the implants [42–44, 72], we will systematically investigate the amount of positive macrophages and the chemotactic activity for inflammatory cells around our device.

## Electronic supplementary material

Supplementary Figure 1
