# Supplementary figures and images for: An important step towards a prevascularized islet microencapsulation device: in vivo prevascularization by combination of mesenchymal stem cells on micropatterned membranes
Source: J Mater Sci Mater Med. 2018 Nov 9;29(11):174. doi: 10.1007/s10856-018-6178-6 (PMC6244873; doi:10.1007/s10856-018-6178-6)

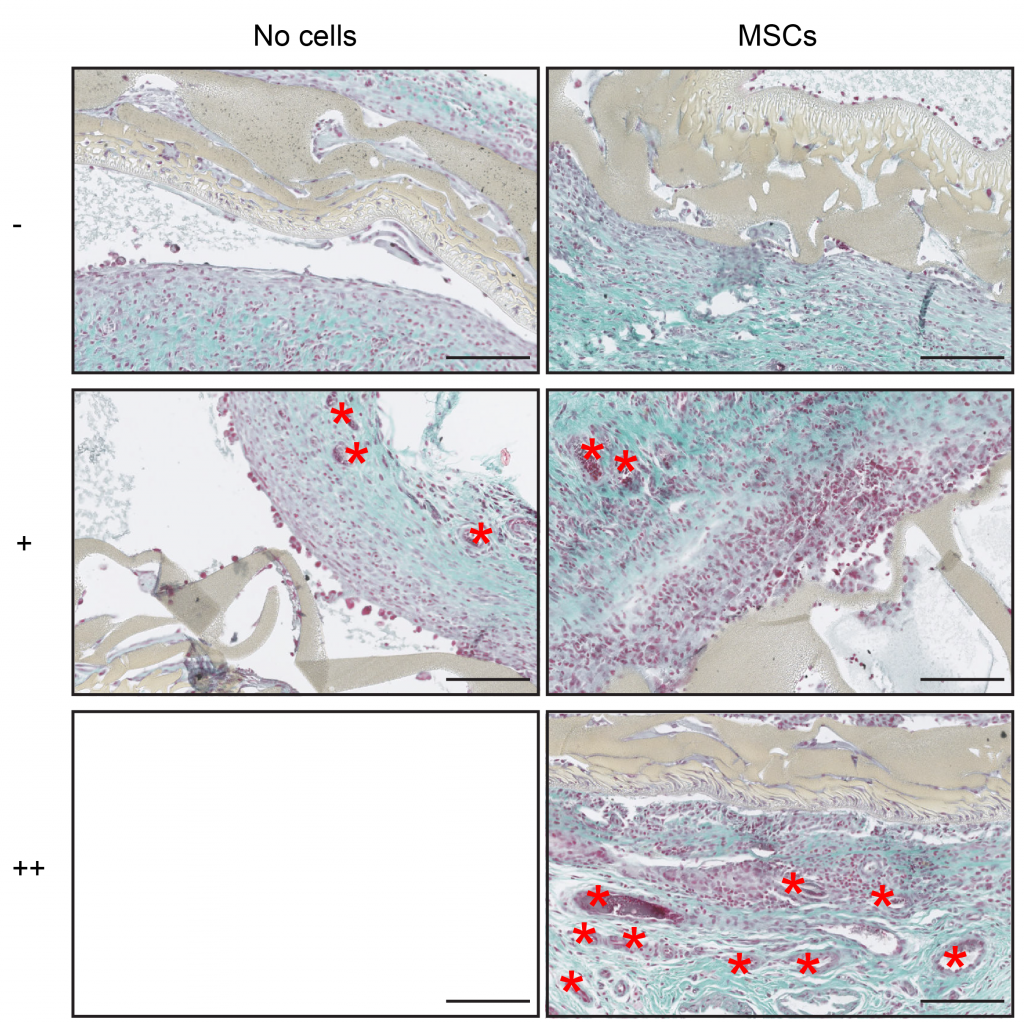

Supplement: Supplementary file 1 — Supplementary Figure 1 [file 10856_2018_6178_MOESM1_ESM.png]
